# Early intervention of atopic dermatitis as a preventive strategy for progression of food allergy

**DOI:** 10.1186/s13223-021-00531-8

**Published:** 2021-03-16

**Authors:** Alyssa Sweeney, Vanitha Sampath, Kari C. Nadeau

**Affiliations:** 1 Sean N. Parker Center for Allergy and Asthma Research at Stanford University, 240 Pasteur Dr. BMI Rm.1755, Palo Alto, CA 94304 USA; 2 Division of Pulmonary, Allergy and Critical Care Medicine, Department of Medicine, Stanford University, Stanford, CA 94305 USA

**Keywords:** Atopic dermatitis, Ceramides, Eczema, Emollients, Filaggrin, Food allergy, Prevention, Skin barrier, Stratum corneum, Trilipids

## Abstract

**Background:**

Atopic diseases, such as atopic dermatitis (AD) and food allergy (FA), have increased in prevalence in industrialized countries during the past few decades and pose a significant health burden. They appear to have a common underlying mechanism and a natural disease progression. AD is generally the first atopic disease to manifest followed by other atopic diseases, such as FA, allergic rhinitis, or allergic asthma suggesting that they are likely different manifestations of the same disease.

**Body:**

Evidence suggests that allergic sensitization occurs through an impaired skin barrier, while consumption of these foods at an early age may actually result in tolerance. This has been termed the Dual-Allergen-Exposure hypothesis. Loss of barrier integrity has been hypothesized to enable penetration of allergens, pollutants, and microbes and initiation of an inflammatory immune cascade of events leading to sensitization. The immune dysfunction is thought to further exacerbate the impaired skin barrier to form a vicious cycle. There is much interest in preventing or protecting the skin barrier from developing a proinflammatory atopic state, which may potentially lead to the development of AD and subsequently, FA.

**Conclusion:**

Research on preventing or treating skin barrier dysfunction is ongoing. A number of studies have evaluated the efficacy of emollients in preventing AD and FA with mixed results. Studies have differed in the study design, population characteristics, emollients type, and frequency, duration, and area of application. Emollient type has varied widely from oils, creams, petrolatum-based lotions, and trilipid creams. Current research is directed towards the use of trilipid emollients that are similar to the skin’s natural lipid composition with a 3:1:1 ratio of ceramides, cholesterol and free fatty acids and a pH that is similar to that of skin to determine their effectiveness for skin barrier repair and prevention of AD and FA.

## Introduction

Allergic diseases, such as atopic dermatitis (AD) and food allergy (FA), have increased in prevalence in industrialized countries during the past few decades and pose a significant health burden. AD is a multifactorial, heterogeneous inflammatory skin disease that affects approximately 13% of children, one third of whom have moderate to severe disease [1]. AD is associated with pruritis, xerosis, lichenification, and eczematous lesions typically on the face (cheeks), neck, arms, and legs. In the US, AD is associated with an adjusted total incremental annual cost between $3,302 and $4,463, depending on the severity of the disease [2]; in Italy, the cost in adults was determined to be €4284 per patient per year [3].

In the US, it is currently estimated that FA affects approximately 1 in 10 adults and 1 in 12 children [4]. Among food-allergic children, a survey found that 42.3% reported ≥ 1 severe FA and 39.9% reported multiple FA [5]. Most allergic reactions are mild; however moderate and severe reactions occur frequently. National time trends show an upsurge of pediatric food-induced anaphylaxis-related hospitalizations and emergency departments visits in the US [6]. Data from a nationwide observational study conducted between 2004 through 2014 in children and adolescents reported 7310 food-induced anaphylaxis related emergency room visits [6]. While any food can cause an allergy, cow’s milk, hen’s egg, peanuts, soy, wheat, tree nuts, fish, and shellfish account for 90% of all FA [7]. Peanut allergy is the most common FA in the U.S. affecting approximately 2% of children [8]. This potentially serious condition usually develops during early childhood and continues into adulthood; approximately 80% of children with peanut allergy will remain peanut allergic throughout their lifetime [9]. FA significantly impacts the quality of life of those affected and their families. In the UK, total costs of peanut allergy were between £33 and 44 million in 2015 [10]; in the US, the overall economic cost of FA was estimated at $25 billion annually or $4184 per affected child per year [11]. Despite efforts of strict adherence to diets void of the offending FA, accidental exposures to food antigens are common due to the numerous possibilities for contamination. The increasing prevalence of atopic diseases and the economic and societal burden they pose presents an unmet and urgent need to develop strategies to reduce the incidence of IgE-mediated food allergies.

Allergic diseases, such as AD and FA, with a Th2-type proinflammatory immune response and increased production of IgE antibodies on exposure to small amounts of otherwise harmless food and environmental allergens are called atopic diseases. Other atopic diseases include allergic rhinitis and allergic asthma. Comorbid atopic diseases in the same individual occur frequently. Studies have found a strong association between AD, particularly AD that is early-onset and severe, and other forms of allergic disease, including FA and asthma [12]. A study found that infants with AD were six times more likely to have egg allergy and 11 times more likely to have peanut allergy by 12 months than infants without eczema [13]. A population-based study found that infants who developed AD within the first year of life were at greater risk for developing FA as confirmed by oral food challenge, within 12 months (one in five infants with AD had FA as compared to one in 25 without AD). Approximately 50% of infants who developed AD within the first 3 months of life requiring use of topical steroids developed FA [14].

Atopic diseases appear to have a natural progression with AD being the first to manifest, generally in infancy or childhood, followed by other atopic diseases, such as FA, allergic rhinitis, or allergic asthma [15]. This natural progression of atopic diseases is termed the “Allergic March” or “Atopic March.” The presence of IgE antibodies, proinflammatory Th2-type cytokines and the natural progression observed with atopic diseases have led some researchers to suggest that they are all different manifestations of the same disease. Researchers have also hypothesized that by preventing or treating AD in early infancy or childhood, it may be possible to prevent its progression to FA. This notion has further been strengthened by evidence of the skin as a route of sensitization for both AD and FA. The skin is thought to be the site of allergic sensitization with AD being the first manifestation of the atopic cascade, followed by a natural progression to FA and other atopic respiratory diseases.

Here we review the mechanism underlying immune dysregulation in atopic disease, current evidence that suggest that dysregulation of the epidermal barrier initiates a cascade of immune events leading to atopic sensitization and increased risk of AD and FA, and risk factors for development of atopic disease. We also review our current knowledge of early intervention to repair or treat skin barrier dysfunction as a strategy for prevention of AD and its progression to FA.

## Immune mechanisms underlying atopic disease

**Fig. 1:**
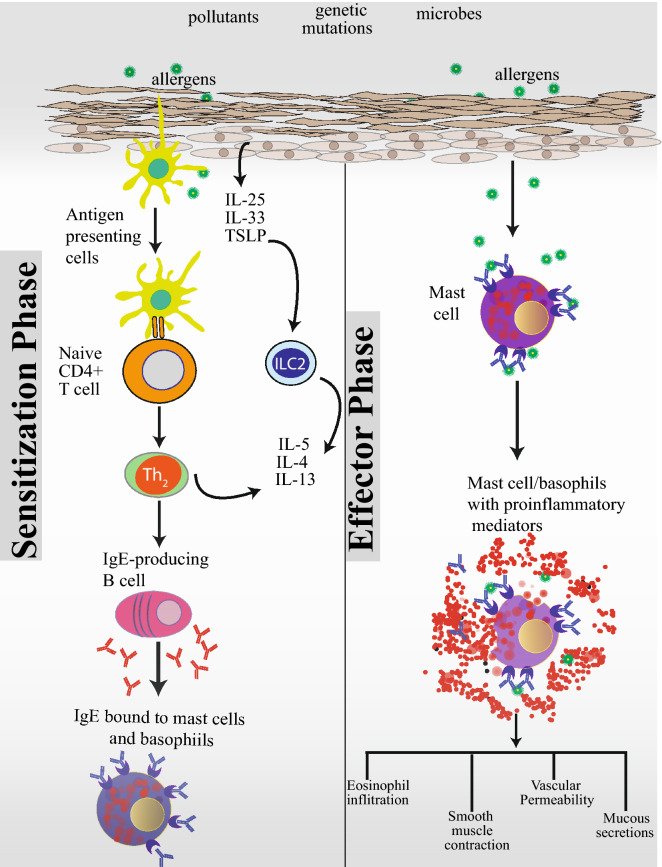
A dysfunctional skin barrier caused by exposure to pollutants, pathogenic microbes, and genetic mutations predisposes towards atopic sensitization, the first manifestation of which is atopic dermatitis. An impaired skin barrier releases proinflammatory epidermal cytokines, IL-25, IL-33, and TSLP. In this proinflammatory environment, resident antigen presenting cells further skew naïve T cells towards a Th2 proinflammatory state. ILC2 cells also play a key role in allergic sensitization. ILC2 cells are found near barrier surfaces and are activated by IL-33. Th2 and ILC2 cells produce a number of cytokines, key among them being IL-4, IL-5 and IL-13. In the presence of IL-4 and IL-13, B-cells undergo isotype class switching to IgE producing cells. IgE antibodies then bind to high-affinity FcεRI receptors on the surface of mast cells and basophils and prime these cells for future encounters with the allergen leading to a state of atopic sensitization. In sensitized individuals, subsequent encounters with an allergen leads to cross linking of IgE bound to FcεRI receptors and degranulation and release of proinflammatory mediators by basophils and mast cells leading to eosinophilic infiltration, smooth muscle contraction, vascular permeability, and mucous secretion

IgE-mediated reactions include two phases—a sensitization phase and an effector phase (Fig. 1). An impaired skin barrier promotes release of proinflammatory epithelial-derived cytokines, IL-25, IL-33, and thymic stromal lymphopoietin (TSLP), which initiate and propagate Type 2 inflammatory responses in AD, food-hypersensitivity reactions, and asthma [16]. These cytokines, initiate a cascade of reactions starting with the activation of dendritic cells and innate lymphoid cells type 2 (ILC2). Dendritic cells, which are present at barrier surfaces capture and present processed allergens through MHC class II molecules to naïve T cells in the draining lymph nodes. Naive T cells in the presence of IL‐4 differentiate into proinflammatory Th2 cells. Th2 cells are the major cell type that skews immune reaction towards allergy by producing cytokines IL-4, IL-5, IL-9, and IL-13. In the presence of IL-4 and IL-13, which are produced by both Th2 and ILC2 cells, B-cells undergo isotype class switching to IgE producing cells and differentiate into plasma cells. IgE antibodies then bind to high-affinity FcεRI receptors on the surface of mast cells and basophils and prime the cells to react on future encounters with the allergen, leading to sensitization.

When primed mast cells and basophils in sensitized individuals encounter an allergen, FcεRI-bound IgE antibodies crosslink and activate degranulation. Degranulation of mast cells and basophils lead to the release of histamine and other inflammatory chemical mediators (cytokines, interleukins, leukotrienes, and prostaglandins) into the surrounding tissue causing several systemic effects, such as vasodilation, mucous secretion, tissue eosinophilic infiltration, and smooth muscle contraction leading to the symptoms of allergic reaction. Symptoms can be mild, moderate, severe, or even life-threatening. Further details of the mechanisms underlying atopic disease can be found in a number of excellent reviews [17].

## Epidermal barrier and immune dysfunction: the inside-out, outside-in, and ouside-inside-outside hypothesis

Evidence suggests that allergic sensitization occurs through an impaired skin barrier (either through genetic defects, mechanical injury, or exposure to chemicals or toxins). Loss of barrier integrity has been hypothesized to enable penetration of allergens, pollutants, and microbes and initiation of an inflammatory immune cascade of events leading to sensitization [18]. Studies using murine models have found that repeated skin exposure to egg allergen induced eczematous skin lesions and asthma-like airway hyperresponsiveness following subsequent exposure to egg allergens aerosol [19]. In mice, exposure to peanut allergens via the application of peanut oil through a disrupted skin barrier (via tape stripping) increased total serum IgE [20]. In a study of around 1,900 infants, skin barrier dysfunction, as determined by transepidermal water loss (TEWL), predicted FA at 2 years of age, supporting the concept of transcutaneous allergen sensitization, even in infants who do not have AD [21]. The increased risk of peanut sensitization and FA has been associated with environmental exposure to peanut allergens. The amount of peanut consumed within the home is associated with the level of peanut allergen in household dust and bedding. [22] Increased exposure to peanut in households is associated with increased peanut sensitization and allergy in children with an impaired skin barrier [23] and AD [24], but not in children without these risk factors, suggesting that sensitization to environmental allergens occurs via an impaired skin barrier. The effect of environmental peanut allergen exposure on children with skin barrier dysfunction provides evidence that sensitization to environmental allergens occurs through the skin. Data from the Avon Longitudinal Study of Parents and Children, a geographically defined cohort study of 13,971 preschool children, suggested that sensitization to peanut protein may occur in children through the application of peanut oil to inflamed skin [25].

Gene mutations that increase skin barrier integrity have also been implicated in AD and FA. Twin studies of AD indicate a heritability of approximately 75% [26] with the remaining 25% attributed to environmental factors. The strongest known genetic risk factor for AD is a loss of function mutation of the gene encoding filaggrin (FLG) [27]. FLG aggregates keratin filaments and is crucial for maintaining skin barrier function. A loss of function mutation of FLG leads to epidermal barrier dysfunction making the skin more permeable to environmental allergens and pollutants, while increasing TEWL. Individuals with double allele mutations in FLG tend to develop early-onset and more severe AD [28]. A study in infants found that by 3 months of age, FLG mutations were associated with AD, dry skin, and increased TEWL. TEWL was elevated even in unaffected FLG mutation carriers suggesting that skin barrier impairment precedes clinical AD [29].

Evidence suggests that both epidermal barrier dysfunction, as well as immune deviation towards a Th2 proinflammatory profile, play important roles in allergic disease. However, whether epidermal barrier dysfunction is secondary to immune deviation (the inside-out hypothesis) or whether immune deviation is secondary to a dysfunctional epidermal barrier (the outside-in hypothesis) has been debated. The discovery of FLG mutation and its role in epidermal integrity and AD provided strong evidence of the outside-in hypothesis. Dupilumab, an anti-IL4R-monoclonal antibody (which blocks the action of IL-4 and IL-13) is approved for the treatment of AD. Similarly, omalizumab, an anti-IgE antibody is approved for the treatment of allergic asthma. These antibodies which ameliorate symptoms of AD and allergic asthma by targeting type-2 immune responses lend support for the inside-out hypothesis. The most recent hypothesis being put forth is the “outside-inside-outside” view, which suggests that the skin barrier deficiency leads to immune imbalance, which further exacerbates the impaired skin barrier to form a vicious cycle [30].

## Environmental factors that influence epidermal barrier integrity and increase risk of AD and FA

With urbanization, barrier surfaces are increasingly exposed to air pollutants, which have been shown to affect skin barrier integrity and increase risk of atopic diseases. Increased hygiene has led to decreases in exposure to microbial diversity. In recent years, numerous studies have shown the importance of the gut and skin microbiome in maintaining immune health and barrier integrity. Factors associated with a healthy microbiome include vaginal delivery, breast feeding, and presence of older siblings. Dysbiosis of the microbiome is associated with Caesarean section, increased use of soaps and detergents, processed foods including formula milk, and exposure to antibiotics and antacids [31].

A study of AD patients found that the severity of AD symptoms was positively correlated with outdoor temperatures, relative humidity, precipitation, particulate matter (PM) with an aerodynamic diameter < 10 μm (PM_10_), NO_2_, ozone, and total pollen count [32]. Short-term exposure to PM was found to exacerbate AD in young children living in an industrial urban area. The study also found that PM_2.5_ had a stronger effect than PM_10_ on exacerbation of AD symptoms [33]. The study of 14,614 children from the Seoul Atopy Friendly School Project Survey in Seoul, Korea, in 2010 found increased risk of eczema was associated with NO_2_ and PM_10_ [34].

Studies indicate that low pH values in skin extracellular spaces play an important role in the regulation of enzyme activity, especially in keratinization and barrier regeneration [35]. Soaps and detergents have been implicated in increasing skin pH, enhancing the activity of proteases, decreasing the activity of enzymes associated with lipid synthesis, and disrupting epidermal integrity and cohesion [36]. In mice models of AD, antibiotic use was associated with increased TEWL compared to those treated with probiotics [37]. A large retrospective cohort study found that the use of acid-suppressive medications and antibiotics during the first 6 months of infancy was associated with subsequent development of allergic disease [38]. *Clostridium difficile* colonization during infancy has been associated with a higher risk of developing allergic diseases during early childhood [39]. In AD, colonization by *Staphylococcus aureus* in skin is commonly observed and has been correlated with increased expression of proinflammatory cytokines, IL-4, IL-13, IL-22, and TSLP [40].

## Prevention of food allergy

Palforzia, a peanut allergen formulation, is currently the only FDA approved oral immunotherapy treatment for FA. For other food allergens, there are no approved treatments and standard of care remains avoidance of allergenic foods and acute management of allergic reactions with antihistamines or epinephrine. Allergen avoidance is difficult to accomplish as many allergenic foods are common ingredients in many foods. A study found that around 58% of individuals with peanut allergy accidentally consume peanuts within a 5-year period [41].

## Oral tolerance: early introduction of allergic foods

Early guidelines for prevention of FA recommended delaying the introduction of allergenic foods to infants to 1–3 years of age [42]. However, even with the implementation of these new guidelines, rates of FA continued to increase. In 2015, results of the LEAP study were published [43]. The LEAP study was a large study of 640 infants between 4 through 11 months of age, randomized to various treatment arms. All of the infants had severe eczema, egg allergy or both. The study found that peanut avoidance was linked to a higher rate of peanut allergy compared to peanut consumption and that regular exposure resulted in a 11.8% decrease in the cumulative incidence of FA. The study provided evidence that early oral introduction of peanuts could prevent allergy in infants at high risk of allergy. The EAT study [44], randomized 1300 exclusively breastfed 3‐month‐old infants from the general population and compared the effect of early introduction of 6 common childhood food allergens (milk, egg, peanut, sesame, fish, and wheat) with exclusive breastfeeding until approximately 6 months of age. Overall, the study found that FA was lower in the group introduced to allergenic foods early but the difference was not statistically significant. As these newer findings did not support avoidance for prevention, guidelines were revised and in 2010 recommended introduction of allergenic foods, such as peanut, tree nuts, and egg at 4 to 6 months of age, after a period of exclusive breastfeeding [45]. Additionally, further analysis of the EAT study in 2019 also supported these guidelines as statistical differences were observed in specific groups of infants at high risk of developing FA: those sensitized to egg or to any food at enrollment and those with eczema of increasing severity at enrollment as compared to exclusively breastfed infants [46]. These studies led to the concept of oral ingestion of allergenic foods as a route to tolerance. This, in conjunction with current understanding of skin barrier defects as a route of atopic sensitization has led to the Dual-Allergen-Exposure hypothesis, which states that exposure to food allergens through the skin can lead to allergy, while consumption of these foods at an early age may actually result in tolerance. However, induction of tolerance through early introduction of food allergens presents a number of challenges [47, 48]. The protective effects of early food protein allergen exposure appear to be allergen-specific suggesting that early introduction of one food protein allergen does not prevent the development of FA to other allergens and that multiple food introductions in early life are necessary to prevent all FA. Another limitation of early allergenic food introduction as an approach to prevent multiple food allergies is the narrow window of time allowed for tolerance induction. Further, adherence presents logistic challenges in the inclusion of multiple allergenic foods in young infants is difficult.

## Emollients for AD prevention and progression to FA

Current evidence suggests that skin barrier defects increase risk of AD, FA, and other atopic diseases. A study by Kelleher et al. found that impairment of skin barrier function, as determined by TEWL, at birth and at 2 months precedes clinical AD [49]. In another study, Kelleher found that neonatal skin barrier dysfunction predicts FA at 2 years of age [21]. In 2014, two randomized controlled trials evaluated the use of emollients in newborn infants at high risk of AD. The study by Simpson, et al. found that daily full body emollient use significantly reduced the cumulative incidence of atopic AD corresponding to a relative risk reduction of 50% [50]. These results were supported by a study by Horimukai, et al., where emollient use lowered AD by 32% [51]. Regular emollient use in newborn infants was also found to decrease diaper dermatitis. The study also measured face TEWL and body stratum corneum hydration (SCH) and found that while TEWL decreased, SCH increased providing further evidence that moisturizing skincare may prevent newborns' diaper dermatitis by improving skin barrier function. The PEBBLES pilot study in 2018 was the first to evaluate the role of emollients in preventing food sensitization in addition to AD. Emollient use demonstrated a non-significant trends towards decreased food sensitization at 6 and 12 months of age in addition to a non-significant trend towards a reduction in AD at 12 months. Per protocol analyses (only including infants who received ≥ 5 days/week of study treatment) revealed a significant reduction in food sensitization at 12 months in the treatment group [52]. McClanahan et al. conducted a 2-year randomized controlled trial of newborns at high risk of AD who received full body application of emollients started with 21 days of birth. In addition to AD, measures of skin barrier integrity and skin microbiome were assessed. The study found a decreasing trend in AD in the intervention group compared to controls at 2 years, but other measures of skin barrier function (TEWL, pH, stratum corneum integrity) or microbiome did not differ between the 2 groups [53]. These early studies showed promise and were followed by a larger randomized controlled study in 2019 by Dissanayake et al. (n = 549) [54]. The study evaluated skin emollients as well as synbiotics (*Bifidobacterium bifidum* OLB6378 plus fructo-oligosaccharides) and consisted of 4 groups: Synbiotics plus skincare, synbiotics only, skincare only, and a control group. The study evaluated food sensitization and AD at 1 year and found that neither synbiotics nor emollients decreased AD or sensitization to food or aeroallergens. Two additional large randomized controlled studies in 2020, the BEEP [55] (n = 1394) and the PreventADALL [56] (n = 2397) study similarly did not find differences between the control group and the emollient intervention group in decreasing incidence of AD. In addition, the BEEP study found no differences in rate of FA or food sensitization (milk, egg, or peanut), allergic rhinitis, wheeze, or aeroallergens at 2 years. The lack of positive results from the Dissanayake, et al., the BEEP, and the PreventADALL studies were surprising and disappointing in light of the promising results of earlier studies.

In the PreventADALL study, the role of skin emollients as well as food intervention was evaluated to see if they would have a synergistic effect in reducing AD. The study had 4 arms—a skin emollient group, a food intervention group, a skin emollient plus food intervention group, and a control group. The study found no benefits of food intervention or emollient in reducing AD. A major difference between studies are the emollients used, and it is now hypothesized that these differences may be due to the type of emollient used. The emollients used in the above studies have varied widely (Table 1) and have included both nonlipid or lipid emollients, which are likely to vary in effectiveness in preventing skin barrier dysfunction or repairing skin barrier integrity. Emollients vary with respect to pH and ability to hydrate the skin. Studies now suggest that trilipid creams may be the most effective as they are very similar to the skin’s natural lipid composition with a 3:1:1 ratio of ceramides, cholesterol, and free fatty acids and a pH that is similar to that of skin [57]. In a recent study, in infants under 10 weeks of age with dry skin or AD treated with a daily trilipid cream for 12 weeks, increases in immune parameters (total IgG4/total IgE) indicative of a decrease in atopic sensitization were observed [58]. In the same cohort, reduced TEWL was also observed [59]. The emollients used in the BEEP and PreventADALL studies were petrolatum based, which are now thought to be less effective than trilipid emollients in reducing TEWL. The only study that has used a trilipid emollient is the pilot study by Lowe, et al. (n = 77), which showed a trend towards reduced risks of AD and food sensitization in infants at 6 and 12 months [52]. Other factors that differed between these studies and which may account for the discrepancy between study results include risk of atopic sensitization, duration, frequency, and site of emollient application. The study by Dissanayake et al. [54] and PreventADALL [56] included infants from the general population, while earlier pilot studies selected a population at high risk for AD. Duration of intervention and evaluation of incidence of AD varied from 6 months [50], 8 months [51], 12 months [53–56], or 24 months [53, 55]. Frequency of application ranged from 4 times a week [56], daily, [55] to twice daily [52] with whole body application [53] or primarily on the face (cheeks and perioral area) [54]. Taken together, it is highly possible that variations in clinical study designs play a key role (or are a key factor) in the outcome of clinical trials and discrepancy of observed results.

**Table 1:** Randomized trials of emollients for prevention of AD

Study	Year	Study design	Treatment/intervention	Population	Primary endpoints	Results
Simpson et al. [50]	2014	RCT (n = 124). Treated (n = 64), untreated controls (n = 60)	Various emollients: sunflower seed oil or double-base gel or liquid paraffin or Cetaphil cream or Aquaphor healing ointment Application: Full body once a day starting within 3 weeks of birth for 6 months	Newborns at high risk for AD*	Cumulative incidence of AD at 6 months	AD: Statistically significant decreased risk of AD (relative risk reduction of 50%) P = 0.017
Horimukai, et al. [51]	2014	RCT (n = 118);. Intervention group (n = 59), control group (n = 59)	Emulsion-type moisturizer (2e [Douhet] emulsion; Shiseido, Tokyo, Japan). Daily full body application from first week of life for 32 weeks	Newborns at high risk for AD*	Cumulative incidence of AD and eczema at 32 weeks	AD: At week 32, AD decreased by 32% (P = 0.012) in neonates who received emulsion-based moisturizer
Yonezawa et al. [60]	2018	RCT (n = 227). Intervention group (n = 113), control group (n = 114)	Pigeon Baby Milk Lotion [Pigeon] or Atopita Milky Lotion© [Tampei Pharmaceutical, Tokyo, Japan]. One or more applications per day for 12 weeks	Newborns	Incidence of diaper dermatitis, skin barrier function (TEWL, stratum corneum hydration, skin pH, and sebum secretion), and skin problems (redness, erythema, dryness and breakdown) at 1 and 3 months	Compared with the control, the intervention group had significantly lower face TEWL (14.69 ± 7.38 vs 17.08 ± 8.26 g/m [2] per h, *P* = 0.033), and higher face and body stratum corneum hydration (60.38 ± 13.66 vs 53.52 ± 14.55,. *P* = .001) and higher body SCH (58.89 ± 12.96 vs 53.02 ± 10.08 *P* < 0.001). Compared with the control, newborns in the intervention group had significantly lower rates of diaper dermatitis between birth and 1 month old (6.3%vs 15.9% *P* = 0.022) and tended to have lower rates of body skin problems between 1 and 3 months (42.1% vs 55.2% *P* = 0.064)
Lowe, et al. (PEBBLES Study) [52]	2018	RCT (n = 77). Intervention group (n = 39), control group (n = 38)	Ceramide-dominant Emollient (EpiCeram®) applied twice daily to whole body for 6 months	Newborns at high risk of AD (family history of atopic disease)	Incidence of AD, food sensitization at 6 and 12 months	Intention-to-treat analysis showed a non-significant trend towards reduced risks of AD and food sensitization at 6 and 12 months, respectively, in the intervention group. Intention to treat analysis showed non-significant trend towards reduced risks of food sensitization at 6 (12.8 vs 22.9 *P* = *.*36) and 12 months (8.8% vs 19.4 *P* = 0.31). Per protocol analyses (only including infants who received ≥ 5 days/week of study treatment) revealed a significant reduction in food sensitization at 12 months in treatment group
McClanahan, et al. [53]	2019	RCT (n = 100). Intervention group (n = 54), control group (n = 46)	Cetaphil® Restoraderm® (Galderma, Baie d’Urfé, Montreal, Canada). Daily full body application started with 21 days of birth through 2 years	Newborns at high risk of AD	Cumulative incidence of AD, filaggrin mutation analysis, skin microbiome analysis, TEWL, skin pH, stratum corneum integrity	Across all clinical end points, a numerical trend was observed in favour of the intervention, although not statistically significant. A decreasing trend in AD, although not statistically significant was observed at 1 and 2 years. There were no significant differences between groups in skin barrier or microbiome assessments
Dissanayake, et al. [54]	2019	RCT (n = 549). Synbiotics plus skincare (group 1; n = 137), synbiotics only (group 2; n = 137), skincare only (group 3; n = 138), no intervention (group 4; n = 137)	Group 1 received a combination of synbiotics and skincare, group 2 received synbiotics only, group 3 received skincare only, and group 4 received no intervention. Skincare product: Locobase® REPAIR Cream (Daiichi Sankyo, Japan). Synbiotics: combination of 0.5 g (7 × 109 CFU/g)of Bifidobacterium bifidumOLB6378 (Meiji Holdings Co. Ltd., Japan) combined with 0.5 g of fructo-oligosaccharides (Meiji Food Materia Co., Ltd., Japan) twice a day Emollients applied 2–3 times a day on cheeks and the peri-oral area. The parents/guardians were allowed to apply the emollient on other parts of the body at their discretion and were not advised for or against it	Newborns	AD, Sensitization to food and/or inhalant allergen at 12 months	Neither the emollient nor the synbiotic showed any effect on reducing the development of AD or sensitization to food or aeroallergens at 1 year of age
Chalmers, et al. (BEEP study) [55]	2020	RTC (n = 1,394). Intervention group (n = 693), control group (n = 701)	Doublebase Gel® (Dermal Laboratories Ltd.) or Diprobase Cream® (Merck Sharp & Dohme Ltd.). Daily application for 12 months	Newborns at high risk of AD	Incidence of AD at 12 and 24 months	There were no differences in AD, food allergy or food sensitization (milk, egg, peanut), at age 2 between the control group and the intervention group. Similarly, there was no difference in other allergens (grass pollen, cat dander, or dust mite) or incidence of wheeze or allergic rhinitis at age 2
Skjerven, et al. (Prevent ADALL Study) [56]	2020	RCT (n = 2,397). Controls (n = 596), skin emollient group (n = 575), food intervention group (n = 642) skin and food intervention group (n = 583)	Ceridal (GlaxoSmithKline Consumer Healthcare) Early complementary feeding consisted of peanut, cow’s milk, wheat, and egg. Application at least 4 times a week	Newborns	Incidence of AD at 12 months	No statistically significant difference in AD between the control and interventional group. Neither skin emollients nor early complementary feeding reduced development of AD at 12 months

^*^Newborns (0–3 months of age) at risk for AD are defined as having a parent or full sibling who has (or had) physician diagnosed AD, asthma, or allergic rhinitis

## Summary

Evidence for the role of barrier defects and skin barrier dysfunction in atopic diseases is accumulating. There is much interest in preventing or protecting the skin barrier from developing a proinflammatory atopic state, which may potentially lead to the development of AD and subsequently, FA. Over the past few decades, we have gained a better understanding of the molecular mechanisms underlying FA and some of the common mechanism underlying all atopic diseases. Immunotherapy for peanut allergy is currently the only FDA-approved treatment available for peanut allergy. Avoidance of allergenic foods is the current standard of care for those diagnosed with FA with acute management of allergic reactions with antihistamines or epinephrine. A number of treatments, such as vaccines and biologics are in clinical trials for FA. There is much interest in finding ways to prevent AD and other atopic diseases. Current studies on emollients have shown mixed results and further research is warranted.

## Data Availability

Not applicable.

## Abbreviations

AD: Atopic dermatitis
BEEP: Barrier Enhancement for Eczema Prevention
CDC: Center for Disease Control and Prevention
CI: Confidence interval
EAT: Enquiring About Tolerance
FA: Food allergy
FLG: Filaggrin gene
IgE: Immunoglobulin E
IL: Interleukin
ILC2: Innate lymphoid cells type 2
LEAP: Learning early about peanut allergy
MCH: Major histocompatibility complex
NO2: Nitrogen dioxide
PM: Particulate Matter
PEBBLES: Prevention of Atopic Dermatitis by a Barrier Lipid Equilibrium Strategy
PreventADALL: Preventing Atopic Dermatitis and Allergies in Children
SPT: Skin prick test
SCH: Stratum corneum hydration
RCT: Randomized controlled trial
TEWL: Transepidermal water loss
Th: T helper cells
TSLP: Thymic stromal lymphopoietin
UK: United Kingdom
US: United States
